# A case report of fatal COVID-19 complicated by rapidly progressive sepsis caused by *Klebsiella variicola*

**DOI:** 10.1186/s12879-023-08128-9

**Published:** 2023-03-29

**Authors:** Rimi Tanii, Sohei Harada, Hiroki Saito, Koh Okamoto, Yohei Doi, Masahiro Suzuki

**Affiliations:** 1 Department of Emergency and Critical Care Medicine, St. Marianna University Yokohama Seibu Hospital, 1197-1, Yasashi-cho, Asahi-ku, Yokohama, Kanagawa Japan; 2 Department of Infection Control and Prevention, The University of Tokyo Hospital, Tokyo, Japan; 3 Department of Infectious Diseases, The University of Tokyo Hospital, Tokyo, Japan; 4 Department of Microbiology, Fujita Health University School of Medicine, Toyoake, Aichi Japan; 5 Department of Infectious Diseases, Fujita Health University School of Medicine, Toyoake, Aichi Japan; 6 Division of Infectious Diseases, University of Pittsburgh School of Medicine, Pittsburgh, PA USA

**Keywords:** COVID-19, *Klebsiella variicola*, *Klebsiella pneumoniae* complex, Whole genome sequencing, Case report

## Abstract

**Background:**

There is a growing interest in *Klebsiella variicola* as a causative pathogen in humans, though its clinical features and the impact of co-infection or secondary infection with COVID-19 remain unknown.

**Case presentation:**

A 71-year-old man presented with fever, altered mental status and generalized weakness and was admitted to ICU due to severe COVID-19 pneumonia. He was newly diagnosed with type II diabetes mellitus upon admission. On hospital day 3, his respiratory status deteriorated, requiring invasive mechanical ventilation. On hospital day 10, superimposed bacterial pneumonia was suspected and subsequently, broad-spectrum antibiotics were administered for the associated bloodstream infection. On hospital day 13, despite administration of active antibiotics and appropriate source control, he decompensated and died.

The causative organism isolated from blood cultures was initially reported as *K. pneumoniae*, but it was identified as *K. variicola* by a genetic analysis. A representative isolate (FUJ01370) had a novel multilocus sequence typing allelic profile (*gapA*-infB-*mdh*-*pgi*-*phoE*-*rpoB*-*tonB*: 16-24-21-27-52-17-152), to which sequence type 5794 was assigned (GenBank assembly accession: GCA_019042755.1).

**Conclusions:**

We report a fatal case of respiratory and bloodstream infection due to *K. variicola* complicating severe COVID-19. Co-infection or secondary infection of *K. variicola* in COVID-19 is likely under-recognized and can be fulminant as in this case.

## Background

Co-infections or secondary infections with bacteria is uncommon among novel coronavirus disease 2019 (COVID-19) patients, seen in 1% of all patients according to a UK national surveillance study [1]. Nevertheless, bacterial infections are more frequent in critically ill COVID-19 patients, many of which occur as hospital-acquired infection (HAI) [2]. Pneumonia and bloodstream infections are common types of HAIs, and *Klebsiella pneumoniae* is one of the most common causative organisms along with *Staphylococcus aureus* and *Pseudomonas aeruginosa* [1–4]. Among the phylogroups of *K. pneumoniae* complex, *K. pneumoniae* is the most concerning, with hypervirulent strains described in Asian countries in association with severe, pyogenic infections, as exemplified by a fatal case of COVID-19 complicated by respiratory tract infection and bacteremia caused by hypervirulent *K. pneumoniae* sequence type (ST) 86 [5]. While *Klebsiella variicola* has been recently recognized as an important human pathogen, the clinical characteristics and impact of co-infection or secondary infection among COVID-19 patients remain unclear [6].

Here, we present a case of fatal secondary infection caused by *K. variicola* that complicated the clinical course of severe COVID-19.

## Case presentations

In May 2021, a 71-year-old man presented with fever, altered mental status and generalized weakness. He had had fever over 38.0 °C for 4 days prior to admission and had a positive SARS-CoV-2 PCR test at a primary care clinic. He had no known past medical history but was newly diagnosed with type II diabetes mellitus (hemoglobin A1c of 10.3%) upon admission. His vital signs included body temperature of 40.0 °C, blood pressure of 155/86 mmHg, pulse rate of 100 beats/min, respiratory rate of 35 breaths/min, and oxygen saturation of 85% with ambient air. Computed tomography scan of the chest showed bilateral ground-glass opacities and consolidations, consistent with COVID-19 pneumonia. Initial laboratory data showed white blood cells count of 4000/μL, C-reactive protein of 14.14 mg/dL (reference, 0.00–0.14).

He was admitted to intensive care unit and received dexamethasone (6.6 mg/day), remdesivir, and intravenous unfractionated heparin. He subsequently required high flow nasal cannula. No empiric antibiotics were given as no bacterial infection was suspected on admission. Initial bacterial cultures of blood, sputum and urine were all negative.

**Fig. 1:**
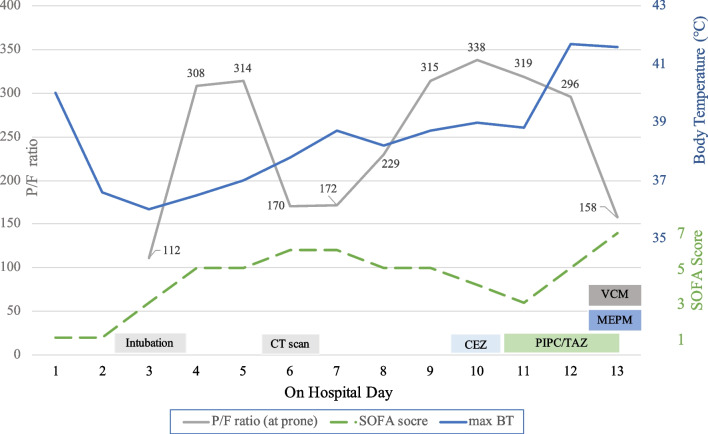
Clinical parameters of the COVID-19 case with *K. variicola*. *SOFA* sequential organ failure assessment, *Max BT* maximum body temperature in a day, *P/F ratio* (partial pressure of arterial oxygen, PaO_2_/fraction of inspired oxygen, FiO_2_, %) ratio, *CT* computed tomography, *CEZ* Cefazolin, *PIPC/TAZ* Piperacillin/Tazobactam, *VCM* Vancomycin, *MEPM* Meropenem

**Fig. 2:**
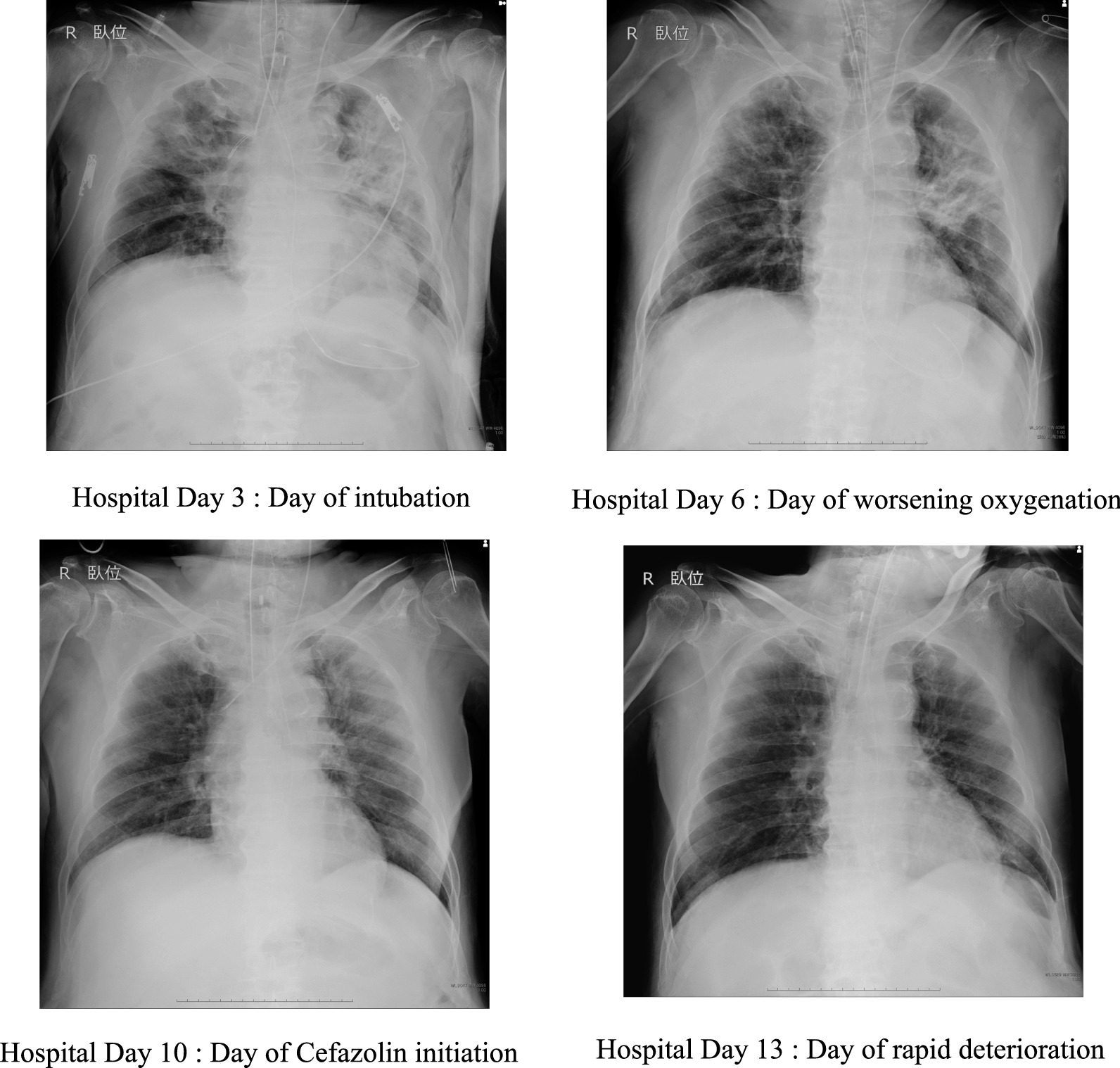
The serial chest radiographs of the patient

On hospital day 3, his respiratory status deteriorated and he was intubated (Figs. 1, 2). On hospital day 6, blood and sputum cultures were repeated due to fever but the blood cultures revealed no growth. His fever persisted and sputum cultures were repeated on hospital day 8. Gram stain of the sputum samples collected on hospital days 6 and 8 revealed Gram-positive cocci (GPC) in clusters. GPC was becoming more predominant and seen within phagocytes in the sample on hospital day 8. The sputum cultures on hospital days 6 and 8 subsequently grew methicillin-susceptible *Staphylococcus aureus* (MSSA) and *Klebsiella pneumoniae* complex. Superimposed MSSA pneumonia was suspected and cefazolin was started on hospital day 10.

On hospital day 11, cefazolin was switched to piperacillin–tazobactam. On hospital day 12, the blood culture taken on hospital day 11 grew Gram-negative rods. All the intravenous catheters were replaced. On hospital day 13, he rapidly developed hypotension, worsening oxygenation and lactic acidosis (Fig. 1). The serial chest radiographs remained unchanged between hospital days 10 and 13 (Fig. 2). Despite prompt interventions including escalation of antibiotics to vancomycin and meropenem, he died on the same day.

Subsequently, blood and sputum cultures collected on hospital days 11, 12 and 13 all grew *K. pneumoniae* complex identified using MicroScan (Beckman Coulter, CA, USA). All isolates were susceptible to cefazolin, piperacillin–tazobactam, meropenem, sulfamethoxazole-trimethoprim, levofloxacin and fosfomycin except the isolates from sputum cultures collected on hospital days 8 and 13 were intermediate to fosfomycin.

The isolates from the sputum cultures collected on hospital days 6, 12 and 13, the blood cultures collected on hospital days 12 and 13, and the culture of the central venous catheter removed on hospital day 12 were subjected to molecular characterization. All isolates were identified as *K. variicola* by a multiplex PCR protocol, which distinguishes the phylogroups of the *K. pneumoniae* complex based on polymorphisms of the SHV-type β-lactamase gene [7]. Another multiplex PCR, which examines the virulence gene profile, demonstrated that all strains carried *kfu*, *entB*, and *mrkB*. These results suggested that the isolates recovered from different body sites on different dates were clonal *K. variicola* isolates. A representative isolate (FUJ01370), which was an isolate from the blood culture on hospital day 12 was further analyzed with whole-genome sequencing by using NextSeq 2000 (Illumina) as described previously [6], and genetic features were characterized using the Pathogenwatch website (https://pathogen.watch/). The capsular genotype and O locus type were determined as KL11 and O3, respectively. The isolate carried a gene for LEN-type β-lactamase (*bla*_LEN-16_), which is a characteristic genetic feature of *K. variicola* [8], and *oqxA*, *oqxB*, *fosA* as antimicrobial resistance genes. Replicons for IncFIB (pNDM-Mar) plasmid, FIA (pBK30683) plasmid, and IncHI1B (pNDM-MAR) plasmid were also identified. The isolate had a novel multilocus sequence typing allelic profile (*gapA*-infB-*mdh*-*pgi*-*phoE*-*rpoB*-*tonB*: 16-24-21-27-52-17-152), to which sequence type 5794 was assigned (GenBank assembly accession: GCA_019042755.1).

## Discussion

We report a case of fatal respiratory and bloodstream infection of *K. variicola* complicating severe COVID-19. The causative organism was initially reported as *K. pneumoniae*, but genetic analysis revealed that it was indeed *K. variicola*. *K. variicola* have been reported among patients with immunocompromising conditions such as malignancy and diabetes [6], and a recent study in Czech Republic reported that the percentage of *K. variicola* as an etiological agent causing nosocomial infections increased significantly (from 1 to 6%, p = 0.0004) in COVID-19 patients compared to the pre-COVID-19 period [9]. But the impact of co-infection or secondary infection among COVID-19 patients remain uncertain.

*Klebsiella variicola* is a member of the *K. pneumoniae* complex, originally reported as a bacterium in plants, but there have been an increasing number of reports on human infections in recent years. Conventional biochemical methods and automated instruments are unable to distinguish *K. variicola* from *K. pneumoniae*, and genetic analysis is required [8]. A recent study in Japan conducted a whole genome sequencing analysis of 140 strains collected as causative organisms of bloodstream infections and initially identified as *K. pneumoniae* reported that 24 (17.1%) were genetically identified as *K. variicola* [6]. As with hypervirulent *K. pneumoniae*, the epidemiology may vary greatly by geographic region, and secondary infections in COVID-19 patients may also be under-reported.

The pathogenic potential of *K. variicola* remains uncertain. In a single-center study in Sweden, *K. variicola* was a significant risk factor for 30-day mortality [10]. However, whether this resulted from virulent clone(s) representing the majority of *K. variicola* cases at the institution is unknown. Some strains of *K. variicola* show hypermucoviscosity of colonies on agar plates or carriage of cardinal virulence genes (*rmpA*, *rmpA2*, and genes for salmochelin and aerobactin), which are biomarkers of hypervirulent *K. pneumoniae*. However, the relationship between these features and virulence in human is unclear [6, 11].

Although *K. variicola* infection was fatal in our patient, the strains detected were neither highly viscous nor associated with cardinal virulence genes. It is reported that some strains have no known microbiological and genetic characteristics of virulence but are hypervirulent clinically [12, 13], raising the possibility that key virulence determinants are yet to be identified in *K. variicola*. Interestingly, mapping of the contigs of the sequenced strain to the virulence plasmids of *K. variicola* isolates (pKV8917, p15WZ-82_Vir), which were reported to be hypervirulent, revealed a common genetic region spanning approximately 150 kb (data not shown). However, this region does not contain any genes that have been associated with hypervirulence of *K. pneumoniae*, and the significance of this plasmid region is unknown [12, 14]].

## Conclusion

In conclusion, we report a fatal case of *K. variicola* infection complicating severe COVID-19. Co-infection or secondary infection of *K. variicola* with COVID-19 is likely under-recognized and can be fulminant as in this case. Further research is required to better describe the epidemiological, clinical, biological and genetic characteristics of *K. variicola*.

## Data Availability

Not applicable.

## Abbreviations

COVID-19: Coronavirus disease 2019
*K. variicola*: *Klebsiella variicola*
ICU: Intensive Care Unit
HAI: Hospital-acquired infection
MSSA: Methicillin-susceptible *Staphylococcus aureus*
PCR: Polymerase chain reaction
